# COVID-19 Pandemic in a Vulnerable Population: Prevalence and Correlates of Anxiety

**DOI:** 10.3390/bs12010013

**Published:** 2022-01-13

**Authors:** Reham Shalaby, Ejemai Eboreime, Nnamdi Nkire, Belinda Agyapong, Hannah Pazderka, Gloria Obuobi-Donkor, Medard Kofi Adu, Wanying Mao, Ernest Owusu, Folajinmi Oluwasina, Vincent I. O. Agyapong

**Affiliations:** 1 Department of Psychiatry, University of Alberta, Edmonton, AB T6G 2B7, Canada; rshalaby@ualberta.ca (R.S.); eboreime@ualberta.ca (E.E.); Nnamdi.Nkire@albertahealthservices.ca (N.N.); bagyapon@ualberta.ca (B.A.); hannah@ualberta.ca (H.P.); obuobido@ualberta.ca (G.O.-D.); medard@ualberta.ca (M.K.A.); wmao2@ualberta.ca (W.M.); eowusu2@ualberta.ca (E.O.); folajinm@ualberta.ca (F.O.); 2 Global Psychological E-Health Foundation, Edmonton, AB T6G 2B7, Canada; 3 Department of Psychiatry, Faculty of Medicine, Dalhousie University, Halifax, NS B3H 2E2, Canada

**Keywords:** anxiety, trauma, COVID-19, cross-sectional, online survey, Fort McMurray

## Abstract

Background: The COVID-19 pandemic has produced negative mental health outcomes. These effects were more prominent in vulnerable communities that experienced prior similar disasters. The study aimed to examine the likelihood and correlates of anxiety symptoms among Fort McMurray (FMM) residents, during the COVID-19 pandemic. Methods: A cross-sectional online survey questionnaire was applied between 24 April and 2 June 2021, at FMM, to gather sociodemographic, COVID-19, and clinical information, including generalized anxiety disorder (using GAD-7 scale). Results: Overall, 186 individuals completed the survey (response rate 74.7%). Most of the respondents were females (159, 85.5%); above 40 years (98, 52.7%); employed (175, 94.1%); and in relationship (132, 71%). The prevalence of moderate-to-severe anxiety was (42.5%, 71) on GAD-7 self-reported scale. Subscribers who reported that they would like to receive mental health support; have received no family support since COVID-19 declaration; and have lost their job during the pandemic were all more likely to report moderate-to-severe anxiety (OR = 3.39; 95% CI: 1.29–8.88), (OR = 4.85; 95% CI: 1.56–15.03), and (OR = 4.40; 95% CI: 1.01–19.24), respectively. Conclusions: Anxiety levels were high among FMM residents, compared to levels before COVID-19. Clinical and social factors significantly predicted likely anxiety in the Fort McMurray population, during the COVID-19 pandemic. It is imperative that resources are mobilized to support vulnerable communities during the COVID-19 pandemic.

## 1. Introduction

Uncertainty and fear as consequences of SARS-CoV-2 infection have increased, producing profound effects on the individual well-being and mental health worldwide. Higher levels of depression, anxiety, and stress have been observed in community members, including healthcare workers [1,2] students [3,4], seniors [5], and patients with health problems including mental health conditions [3], and COVID-19 infection [5]. According to a recent systematic review and meta-analysis by Salari et al., COVID-19 has threatened the mental health of nearly one-third of the general population [6]. The pooled prevalence of stress, depression, and anxiety was estimated at 29.6%, 33.7%, and 31.9%, respectively, among the general population during the pandemic [6]. These figures were essentially higher among COVID-19 patients, in two recent systematic reviews and meta-analyses by Pashazadeh Kan. et al. and Deng. et al. [5,7]. While natural disasters could, and do, affect different populations, a subset of exposed people usually incur clinically significant mental health symptoms of stress, depression, post-traumatic stress disorder (PTSD), and anxiety [8,9]. The available data indicate that people who closely followed COVID-19 news became more anxious and distressed, particularly when the information was exaggerated or fabricated [10,11].

Emerging evidence from studies on the COVID-19 pandemic or similar disasters, suggests that several factors are implicated in the development of mental health and anxiety symptoms; for example, sociodemographic characteristics, such as age, gender, or receiving inadequate social support have significantly impacted mental health outcomes during such difficult times [5,12,13,14]. Additionally, having an infected family member with COVID-19 or excessive fear of becoming infected were also associated with stress-related symptoms [14,15,16]. Furthermore, having an existing mental health condition such as depression or anxiety may present an additional risk, which may contribute to potential exacerbation of the symptoms or relapsing due to service disruption or the need for isolation [13,14,15,16,17]. The received support during the current pandemic was also a significant protector against mental health symptoms. In a previous research [14], authors reported that people who did not receive family support or limited support were more than five times as likely to develop symptoms of post-traumatic stress disorder, compared to those who received absolute support.

The region of Fort McMurray (FMM) is situated in the north of the province of Alberta in Western Canada. By May 2021, the region of FMM had experienced three major disasters over five consecutive years. These include the wildfires associated with an evacuation order in 2016, and flooding and COVID-19 in 2020. Such traumatic experiences are believed to have adversely impacted the mental health and general wellbeing of survivors [9,18]. The one-month prevalence of anxiety symptoms [19], PTSD [20], and depressive symptoms [21] were estimated at 19.8%, 12.8%, and 14.8%, respectively, six months after the wildfire [19]. Compared to a control population, the prevalence of mental health symptoms remained high among adolescents 18 months after the wildfire [18,22]. In 2020, the flooding affected over 1200 structures, costing damages worth $375 million [23]. The physical and economic cost of these experiences is often accompanying emotional difficulties and stress [22,23].

Given the aforementioned information, it is generally expected that there will be a rise in mental health problems due to the burden posed by these traumatic experiences. It is unclear, however, how prevalent the mental health symptoms are among FMM population during the COVID-19 pandemic. In this context, the goal of this study was to examine the prevalence and potential correlates of moderate-to-high anxiety during the month of May 2021 when FMM was considered to be a hotspot for the COVID-19 pandemic in Alberta, Canada [24]. Our specific research questions included: (1) what was the prevalence of moderate-to-high anxiety among residents of Fort McMurray during the month of May 2021 when the city was a hot spot for COVID-19 infections? (2) What were the sociodemographic, clinical, and COVID-19-related predictors for moderate-to-high anxiety in residents of Fort McMurray during the peak of the pandemic? We hypothesize that the prevalence of moderate-to-high anxiety symptoms in residents of FMM is higher than the prevalence reported for the general population in Canada and in other jurisdictions during the pandemic. We also hypothesize, that in addition to sociodemographic and clinical characteristics, there would be COVID-19-related variables that are independently associated with moderate-to-high anxiety symptoms in the respondents.

**Figure 1:**
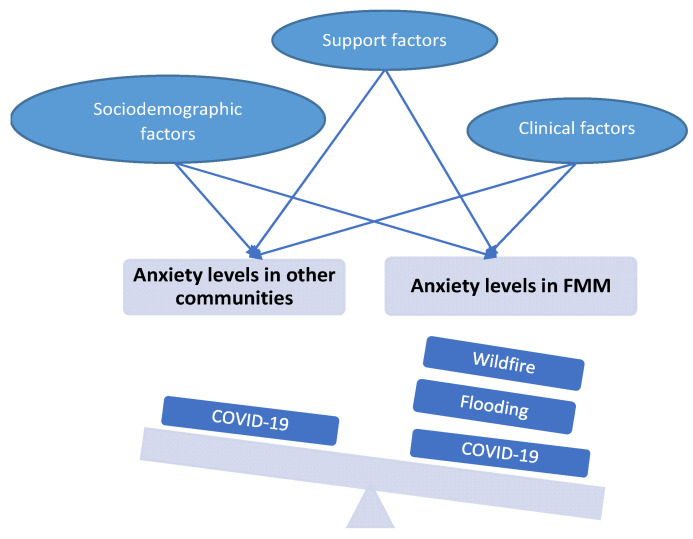
Conceptual framework for the prospective impact of multiple traumas and the predictive factors for high anxiety levels in Fort McMurray (FMM) community versus other communities.

The conceptual framework explaining the anticipated high prevalence of moderate-to-high anxiety in Fort McMurray in comparison to those of the general public in Canada during the pandemic was designed by the study authors and based on empirical evidence that the history of frequent disasters or major traumas may increase levels of mental health conditions [6,9,18,20], and therefore, the anxiety level may be increased under these circumstances and exceeding the levels reported in other communities during the same pandemic (Figure 1). Additionally, the framework suggests a number of potential risk factors that may predispose toward high anxiety levels during the COVID-19 pandemic (Figure 1).

## 2. Materials and Methods

### 2.1. Study Setting and Design

The study data were collected through an online survey completed by people in Fort McMurray (FMM), Canada. FMM represents the urban service area of the Northern Alberta Regional Municipality of Wood Buffalo, with a diverse population of 111,687 as of the 2018 census [25], mainly in temporary project accommodations and in adjoining rural communities [20]. Through a cross-sectional survey introduced from 24 April to 2 June 2021, a self-administered questionnaire was sent to residents of FMM via REDCap [26]. The questionnaire was distributed randomly via email using government, school, occupational, and community platforms. Consent was implied, by completing the survey. Study approval was granted by Alberta Health Research Ethics Committee (Pro00066054).

### 2.2. Outcome Measure

The Generalized Anxiety Disorder-7 (GAD-7) scale was used to assess likely anxiety in the respondents. The scale consists of seven self-reported items that define the symptoms of GAD. Items are rated on a 4-point Likert-type scale (0 = *not at all* to 3 = *nearly every day*). Scores range from 0 to 21 with higher scores indicating more severe GAD symptoms [27]. Patients with a GAD-7 score of 10 or more were deemed to have likely anxiety. Two categories were thus generated: Low anxiety <10 and moderate-to-high anxiety ≥ 10. In a heterogeneous clinical population, the GAD-7 has excellent internal consistency and a one-factor structure [27], indicating that its items are all representative of one construct. The GAD-7 is suggested as a valid tool for screening and assessing the severity of GAD in clinical practice and research [28].

### 2.3. Statistical Analysis

Data were analyzed using SPSS Version 25 (IBM Corp 2011) [29]. Descriptive statistics were administered for demographic, clinical, and COVID-19-related variables based on the relationship status. Chi-squared analysis was run to examine all the variables in relation to the likely anxiety categorical variable (low and moderate-to-high anxiety). Logistic regression analysis was employed to identify significant predictors of likely anxiety. The model included the significant (*p* < 0.05) or near significant (0.1 ≥ *p* ≥ 0.05) variables with the likely anxiety, obtained from the univariate analysis. Odds ratios (OR) and confidence intervals were reported, determining the predictor variables for respondents to self-report likely anxiety during the COVID-19 pandemic, controlling for the rest of the variables.

Correlational analysis was performed to exclude any strong intercorrelations (Spearman’s correlation coefficient from 0.7 to 1.0 or from −0.7 to −1.0) among predictor variables. No imputation was applied, and reported data represent the complete responses.

## 3. Results

Of the 249 individuals who clicked on the survey link, 186 completed the survey, yielding a response rate of 74.7%. Descriptive information of demographic, clinical, and COVID-19-related data on the full sample of patients (N = 186) were examined against the relationship status of the participants (Table 1). Overall, 71% (132) reported that they were in a relationship (married, cohabiting, or partnered) while 29% (54) reported they were not in a relationship (divorced, widowed, or single). From Table 1, most respondents 85.5% (159) were females; 52.7% (98) were above 40 years of age; 94.1% (175) were employed; 78% (145) owned their own homes; 48.4% (90) reported having no mental health diagnosis, while 64.5% (120) reported not receiving psychotropic medications; 38.7% (72) reported receiving mental health counseling; and 52.7% (98) reported they would like to receive mental health counseling.

Regarding COVID-19-related data, during the pandemic, 92% (160) of respondents reported having been fearful about contracting the virus; 96.6% (168) were fearful about their close friends or family members contracting the coronavirus; 72.1% (124) reported having close friends or family members who have been sick from the coronavirus disease; 60.1% (104) had to self-isolate or self-quarantine due to COVID-19 symptoms, recent travel, or because of being in contact with someone who may have COVID-19; 44.3% (77) watched television images of sick and dead people caused by coronavirus, on a daily basis; 58% (101) read newspaper and internet articles related to the pandemic, on a daily basis; 12.1% (21) reported losing their job due to the COVID-19 pandemic; and received sufficient support from family and friends since the COVID-19 pandemic declared. Regarding receiving support since the COVID-19 pandemic was declared, respondents reported receiving a some-to-high level of support from: family and friends were 75.7% (131); from the Government of Canada were 29% (49); from the Government of Alberta were 27% (41); and from the employer were 72.1% (124). Moderate-to-severe anxiety was reported by 42.5% (71) of the respondents.

The univariate analysis in Table 2 included 30 demographic, clinical, and COVID-19-related variables in relation to the likelihood of anxiety. Chi-squared or Fisher exact test revealed significant association between the likelihood of anxiety and 13 variables: employment status; history of depression diagnosis; history of anxiety diagnosis; history of any mental health diagnosis; history of antidepressant medications; history of Benzodiazepine medications; history of receiving any psychotropic medications; receiving mental health counselling; willingness to receiving mental health counselling; losing job due to COVID-19 pandemic; receiving sufficient support from family or friends; receiving sufficient support from Government of Canada; and receiving sufficient support from the employer, since the COVID-19 pandemic declared. No variables showed a near significant association with likely anxiety.

Table 3 illustrates the multivariate logistic regression model employed to predict likely anxiety among study respondents. The model included 10/13 out of the chi-squared predictor variables, after removal of two variables: *history of any mental health diagnosis* and *history of antidepressant medications* that showed high correlation with other variables (r_s_ > 0.7), and another variable: *history of Benzodiazepine medications* that showed no variability.

The model was statistically significant; *Χ^2^* (df = 12; n = 161) = 75.39, *p* < 0.001, suggesting that the model could distinguish between respondents who did or did not exhibit likely anxiety during the COVID-19 pandemic. The model accounted for 37.4% (Cox and Snell R^2^) to 50.1% (Nagelkerke R^2^) of the variance. According to the goodness-of-fit statistic using the Hosmer–Lemeshow goodness-of-fit test, the model was adequately fit (Chi^2^ = 10.0; *p* = 0.27) and correctly classified 75.8% of cases

Only three variables, *the desire to have MH support*, *receiving family support,* and *job loss due to the COVID-19 pandemic* showed significant prediction of likely GAD in the model. Subscribers who reported that they would like to receive mental health support were more than three times likely to express moderate-to-severe anxiety during the COVID-19 pandemic, compared to those who were not willing to receive MH support (OR = 3.39; 95% CI: 1.29–8.88), while controlling for the other variables. Likewise, respondents who reported losing their job during the pandemic were four times more likely to report likely anxiety, compared to those who did not lose their job (OR = 4.40; 95% CI: 1.01–19.24), after controlling for other model variables.

Similarly, the subscribers who reported receiving limited or no family support since the declaration of COVID-19 pandemic were almost five times more likely to report moderate-to-severe anxiety, compared to those who received some-to-high level of family support (OR = 4.85; 95% CI: 1.56–15.03), while controlling for other variables. The largest contribution to the model was provided by the received family support variable (Wald = 7.46).

## 4. Discussion

Our study examined the prevalence and the potential predictors of likely generalized anxiety disorder (GAD) development during the COVID-19 pandemic, among Fort McMurray’s study participants. The prevalence of likely GAD was 42.5% in our study (based on self-reported GAD-7 scale). In comparison with other studies during similar disasters in the same community, the prevalence in our study was overall higher than what was previously reported 6 months and 18 months after the wildfires (20% and 18%, respectively) [19,30]. This may reflect the stringent impact of the pandemic upon mental health and wellbeing. The time of sampling may be an additional factor; where the current survey was carried out during the continuous pandemic when there was still so much uncertainty, while the wildfire surveys were conducted at 6 and 18 months after the wildfires. On the other hand, this prevalence is consistent with other studies of the same variable during the pandemic in different places such as China and other parts of Canada [31,32] in which GAD prevalence was reported at 44.6% and 46.7%, respectively. A lower prevalence of anxiety was reported in a systematic review conducted during the pandemic (27.3–39.6%); however, the study reported a marked discrepancy in such values between females and males at 48% and 28%, respectively [5]. Given that the majority of our respondents were females, the reported high anxiety levels may be related to this particular category. Consequently, it appears that, contrary to our stated hypothesis, the prevalence of moderate-to-high anxiety in FMM, a community with a history of two previous natural disasters, was aligned with and in some consideration, even less than (when considering the female majority of the sample) than the prevalence of moderate-to-high anxiety experienced in other communities during the COVID-19 pandemic. This may be attributed to either the unprecedented effect of the pandemic that triggered the maximum levels of mental distress regardless of the previous traumas, or to some extent, to the adaptability and resilience developed by FMM residents after experiencing frequent traumas.

Among sociodemographic characteristics, employment status showed a significant association with the likelihood of anxiety, where the majority of unemployed people reported moderate-to-high anxiety symptoms; however, the limited number of respondents in this category (n = 9) may impair any meaningful interpretation of such a finding. However, given the fact that the ability to work from home during lockdown is a consideration specific to pandemic trauma (compared to the wildfire or flooding those residents had also experienced), it stands to reason that unemployment would be associated with significant increases in anxiety. Other than employment status, none of the demographic characteristics showed an association with anxiety symptoms during the pandemic. Although females and the younger age expressed a higher prevalence of anxiety symptoms, compared to males and older age groups, respectively, the difference was minimal and not significant. This contradicts other studies, where the younger age groups were found to be more prone to express mental health stresses, including anxiety symptoms during the pandemic, compared to the older age groups [5,12]. The relatively small sample size of our study may not have elicited that difference as reported in other studies.

The three variables that correlated with high odds of likely GAD in our study were the need to receive mental health support, job loss due to the pandemic, and the limited/lack of family support since the declaration of the pandemic. Respondents who reported their need to receive or were receiving mental health support were consistently reported, in other studies, to be at a higher risk of expressing mental health symptoms [14,30]. However, this finding may need to be interpreted cautiously and in the context of the association relationship between the need of support and the underlying pathology. Although this need may be perceived as a serious indicator for a possible presence or development of psychopathology, the reverse may be the case, where the underlying anxiety symptoms may have triggered the excessive MH need, particularly during the pandemic

In the same context, our results did not find underlying mental health conditions to significantly predict the presence of likely anxiety. Although those who reported having a diagnosis of depression or anxiety at the time of the survey were from two to three folds more likely to express anxiety symptoms, these associations were not statistically significant. This contrasts with the previous literature, in which people who had prior mental health conditions were at a high risk of developing mental illness post-disaster [14,33,34]. This may be explained by those who have a well-diagnosed mental health condition may be in a better position than those who are yet to be diagnosed, as receiving regular therapeutic support may, otherwise guard against further symptomatology. However, the timing of data collection in relation to the pandemic phase may also have a role. In one study, for example, the authors found that the pre-existing mental health condition was a significant predictor for psychological distress early in the study phase, while the effect disappeared later in the study [13]. Similarly, they found that people with increasing mental health symptoms have experienced resilience early in the pandemic, which faded later, and in contrast to those who reported high scores in the early weeks, they showed considerable improvement with the pandemic progression [13].

Our results showed that losing a job due to the pandemic represented a significant risk factor of developing anxiety symptoms, compared to those who did not lose their jobs in our study cohort. This finding is consistent with previous research which reported losing a job or job insecurity was linked to various mental health adversities including depression and psychological distress [35,36]. People who lost their job in an Australian cohort were four times more likely to express severe psychological distress compared to the employed people during the pandemic [36]. Furthermore, people who kept working during the pandemic demonstrated better mental and physical health, and life satisfaction, compared to the people who stopped working; these results were fairly similar for those who worked at their home or at their office [37].

In our study, the role of family or friends’ support during the COVID-19 pandemic was a determinant for the likelihood of GAD. Those who reported receiving limited or no support from the family or friends were more prone to likely GAD, compared to those receiving some-to-high level of support. Several studies supported such a finding, exploring the role of the family and the social support in ameliorating anxiety and improving coping and resilience, among other distresses during and after disasters and pandemics [14,19,36,38]. Moreover, having sufficient support prior to a disaster may act as a barrier against psychological trauma developing usually after the crisis [9]. Additionally, having poor relationships or lack of social support, prior to a disaster is accompanied by more psychopathologies [33].

### Limitations of the Study

Our study has several limitations. Firstly, the sample size is relatively small; thus, our results may need to be replicated with other large-scale studies. Secondly, the majority of our respondents were females, which may limit the generalizability of the data. Thirdly, our study results were limited by the frequency of the reported conditions; for example, none of the study participants reported a history of psychotic disorder, on which we could not establish the relationship between the history of psychotic disorder and the present condition of anxiety symptoms. Fourth, given that the survey was shared via e-mail by community partners, it was not possible to specify how many individuals received the survey links. The study response rate was calculated using the number of unique individuals who clicked on the link as the denominator, rather than the number who received the link, as the latter could not be determined, which could lead to an overestimation of the response rate. Finally, the measurement scale for likely GAD was self-reported and was not supported by objective and detailed clinical assessment.

## 5. Conclusions 

This study highlighted the mental health burdens—mainly anxiety—during the COVID-19 pandemic in FMM. It adds to the clinical and research evidence related to the field of trauma and disasters. The study identified the prevalence and the correlates of the likelihood of developing GAD among residents of Fort McMurray, Canada, during the COVID-19 pandemic. Against our expectations, although we found a high level of anxiety experienced among FMM residents during the current pandemic when compared to other previous disasters, this level was fairly aligned with the results of other reports within non-traumatized communities during the COVID-19 pandemic. On the other hand, in line with our hypothesis, the received support from the family and friends and the reported need for mental health support are substantially related to likely GAD, although none of the demographic or underlying clinical conditions could predict likely anxiety. Such results may need cautious interpretation in light of the small sample size and limited reportedly history of clinical conditions. Therefore, it is imperative for the research and policy to mobilize the resources that widely examine and safeguard against developing anxiety symptoms, with a clear understanding of the leading causes that contribute to such outcomes during pandemics, in order to improve personal functionality and achieve a better quality of life.

### Key Points

1. The prevalence of anxiety symptoms among Fort McMurray residents was 42.5% based on GAD-7, a self-reported measure;
2. The subscribers who stated that they would like to receive mental health support were three times more likely to demonstrate likely anxiety;
3. The lack of family/friend support during the COVID-19 pandemic demonstrated a significant risk factor for developing anxiety symptoms;
4. Similarly, losing the job during the COVID-19 pandemic was significantly related to the development of anxiety symptoms.

## Figures and Tables

**Table 1:** Demographic profile, clinical characteristics, and support received by the study population.

Variables	in a Relationship	Not in a Relationship	Total
	n (%)	n (%)	n (%)
**Gender**			
Male	17 (12.9)	10 (18.5)	27 (14.5)
Female	115 (87.1)	44 (81.5)	159 (85.5)
**Age categories**			
≤40y	62 (47.0)	26 (48.1)	88 (47.3)
>40y	70 (53.0)	28 (51.9)	98 (52.7)
**Employment status**			
Employed	125 (94.7)	50 (92.6)	175 (94.1)
Unemployed	7 (5.3)	4 (07.4)	11 (5.9)
**Housing status**			
Own home	113 (85.6)	32 (59.3)	145 (78.0)
Renting	19 (14.4)	22 (40.7)	41 (22.0)
**History of mental health diagnosis from a health professional**			
Depression	37 (28.0)	21 (38.9)	58 (31.2)
Bipolar Disorder	3 (2.3)	3 (5.6)	6 (3.2)
Anxiety	54 (40.9)	24 (44.4)	78 (41.9)
Schizophrenia	0 (0.0)	0 (0.0)	0 (0.0)
Personality Disorder	0 (0.0)	2 (53.7)	2 (1.1)
Other	12 (9.1)	5 (9.3)	17 (9.1)
No mental health diagnosis	69 (52.3)	21 (38.9)	90 (48.4)
**History of psychotropic medications**			
Antidepressants	34 (25.8)	25 (46.3)	59 (31.7)
Antipsychotics	2 (1.5)	2 (3.7)	4 (2.2)
Benzodiazepines	3 (2.3)	1 (1.9)	4 (2.2)
Mood stabilizers	5 (3.8)	7 (13.0)	12 (6.5)
Sleeping tablets	12 (9.1)	9 (16.7)	21 (11.3)
Other	2 (1.5)	1 (1.9)	3 (1.6)
On no psychotropic medication	92 (69.7)	28 (51.9)	120 (64.5)
**Respondents received MH counselling in the past year**	48 (36.4)	24 (44.4)	72 (38.7)
**Respondents would like to receive MH counselling**	66 (50.0)	32 (59.3)	98 (52.7)
**Respondents who have been fearful about contracting the coronavirus during the pandemic**	113 (89.7)	47 (97.9)	160 (92.0)
**Respondents who have been fearful about their close friends or family members contracting the coronavirus during the pandemic**	121 (96.0)	47 (97.9)	168 (96.6)
**Respondents who reported having close friends or family members who have been sick from the coronavirus disease**	94 (75.8)	30 (62.5)	124 (72.1)
**Have you had to self-isolate or self-quarantine due to COVID-19 symptoms, recent travel, or because you were in contact with someone who may have COVID-19?**			
Yes	77 (61.6)	27 (56.3)	104 (60.1)
No	48 (38.4)	21 (43.8)	69 (39.9)
**During the period of the COVID-19 pandemic, how frequently did you watch television images of sick and dead people caused by coronavirus?**			
Daily	58 (46.0)	19 (39.6)	77 (44.3)
Less than daily	53 (42.1)	21 (43.8)	74 (42.5)
Respondents did not watch the TV images of the devastation	15 (11.9)	8 (16.7)	23 (13.2)
**During the period of the COVID-19 pandemic, how frequently did you read newspaper and internet articles related to the pandemic?**			
Daily	72 (57.1)	29 (60.4)	101 (58.0)
Less than daily	50 (39.7)	19 (39.6)	69 (39.7)
Respondents did not read newspaper and internet articles of the devastation	4 (3.2)	0 (0.0)	4 (2.3)
**Did you lose your job due to the COVID-19 pandemic?**			
Yes	14 (11.1)	7 (14.6)	21 (12.1)
No	112 (88.9)	41 (85.4)	153 (87.9)
**Received sufficient support from family and friends since the COVID-19 pandemic declared**			
Some-to-high level of support	94 (75.2)	37 (77.1)	131 (75.7)
Limited or no support	31 (24.8)	11 (22.9)	42 (24.3)
**Received sufficient support from Government of Canada since the COVID-19 pandemic declared**			
Some-to-high level of support	38 (30.9)	11 (23.9)	49 (29.0)
Limited or no support	85 (69.1)	35 (76.1)	120 (71.0)
**Received sufficient support from Government of Alberta since the COVID-19 pandemic declared**			
Some-to-high level of support	32 (29.9)	9 (20.0)	41 (27.0)
Limited or no support	75 (70.1)	36 (80.0)	111 (73.0)
**Received sufficient support from the employer since the COVID-19 pandemic declared**			
Some-to-high level of support	90 (72.6)	34 (70.8)	124 (72.1)
Limited or no support	34 (27.4)	14 (29.2)	48 (27.9)
**Likely anxiety level**			
Low anxiety	73 (60.3)	23 (50.0)	96 (57.5)
Moderate-to-severe anxiety	48 (39.7)	23 (50.0)	71 (42.5)

MH: Mental health.

**Table 2:** Chi-squared test of association between demographic, clinical, and COVID-19 characteristics and likely anxiety.

Variables	Low Anxiety	Moderate-to-High Anxiety	Chi Square/Fisher’s Exact	*p* Value	Effect Size
	n (%)	n (%)			
**Demographic Characteristics**					
**Gender**					
Male	15 (65.2)	8 (34.8)	0.65	0.50	0.06
Female	81 (56.3)	63 (43.8)			
**Age categories**					
≤40y	40 (51.9)	37 (48.1)	1.79	0.21	0.10
>40y	56 (62.2)	34 (37.8)			
**Employment status**					
Employed	95 (60.1)	63 (39.9)	8.37	<0.001 *	0.22
Unemployed	1 (11.1)	8 (88.9)			
**Relationship**					
In a relationship	73 (60.3)	48 (39.7)	1.46	0.29	0.09
Not in a relationship	23 (50.0)	23 (50.0)			
**Housing status**					
Own home	78 (58.6)	55 (41.4)	0.36	0.57	0.05
Renting	18 (52.9)	16 (47.1)			
**Clinical Characteristics**					
**History of depression from a health professional?**					
Yes	19 (35.8)	34 (64.2)	14.87	<0.001 *	0.30
No	77 (67.5)	37 (32.5)			
**History of bipolar disorder from a health professional?**					
Yes	3 (60.0)	2 (40.0)	0.01	0.99	0.01
No	93 (57.4)	69 (42.6)			
**History of anxiety from a health professional?**					
Yes	29 (41.4)	41 (58.6)	12.71	<0.001 *	0.28
No	67 (69.1)	30 (30.9)			
**History of personality disorder from a health professional?**					
Yes	0 (0.0)	1 (100.0)	**	0.43	0.09
No	96 (57.8)	70 (42.2)			
**History of any mental health diagnosis from a health professional?**					
Yes	41 (47.7)	45 (52.3)	6.98	0.01 *	0.20
No	55 (67.9)	26 (32.1)			
**History of antidepressant medications**					
Yes	24 (45.3)	29 (54.7)	4.73	0.04 *	0.17
No	72 (63.2)	42 (36.8)			
**History of antipsychotics medications**					
Yes	2 (66.7)	1 (33.3)	**	0.99	0.03
No	94 (57.3)	70 (42.7)			
**History of benzodiazepines medications**					
Yes	0 (0.0)	4 (100.0)			
No	96 (58.9)	67 (41.1)	**	0.03 *	0.18
**History of mood stabilizers medications**					
Yes	5 (50.0)	5 (50.0)			
No	91 (58.0)	66 (42.0)	0.24	0.75	0.04
**History of sleeping tablets medications**					
Yes	8 (44.4)	10 (55.6)			
No	88 (59.1)	61 (40.9)	1.40	0.31	0.09
**On any psychotropic medication**					
Yes	28 (46.7)	32 (53.3)	4.48	0.05	0.16
No	68 (63.6)	39 (36.4)			
**Respondents received MH counselling in the past year**					
Yes	23 (36.5)	40 (63.5)	18.22	<0.001 *	0.33
No	73 (70.2)	31 (29.8)			
**Respondents would like to receive MH counselling**					
Yes	31 (35.2)	57 (64.8)	37.71	<0.001 *	0.48
No	65 (82.3)	14 (17.7)			
**COVID-19-related Characteristics**					
**Respondents who have been fearful about contracting the coronavirus during the pandemic**					
Yes	86 (56.2)	67 (43.8)	1.22	0.40	0.09
No	10 (71.4)	4 (28.6)			
**Respondents who have been fearful about their close friends or family members contracting the coronavirus during the pandemic**					
Yes	90 (55.9)	71 (44.1)	**	0.39	0.17
No	6 (100.0)	0 (0.0)			
**Respondents who reported having close friends or family members sick from the coronavirus disease**					
Yes	69 (58.5)	49 (41.5)	0.38	0.60	0.05
No	25 (53.2)	22 (46.8)			
**Have you had to self-isolate or self-quarantine due to COVID-19 symptoms, recent travel, or because you were in contact with someone who may have COVID-19?**					
Yes	58 (56.9)	44 (43.1)	0.01	0.99	0.01
No	37 (57.8)	27 (42.2)			
**During the period of the COVID-19 pandemic, how frequently did you watch television images of sick and dead people caused by coronavirus?**					
Daily	37 (50.7)	36 (49.3)	0.25	0.24	0.13
Less than daily	47 (64.4)	26 (35.6)			
Respondents did not watch the TV images of the devastation	12 (57.1)	9 (42.9)			
**During the period of the COVID-19 pandemic, how frequently did you read newspaper and internet articles related to the pandemic?**					
Daily	51 (52.6)	46 (47.4)	**	0.31	0.12
Less than daily	42 (63.6)	24 (36.4)			
Respondents did not read newspaper and internet articles of the devastation	3 (75.0)	1 (25.0)			
**Did you lose your job due to the COVID-19 pandemic?**					
Yes	5 (25)	15 (75)	9.81	<0.01 *	0.24
No	91 (61.9)	56 (38.1)			
**Received sufficient support from family and friends since the COVID-19 pandemic declared**					
Some-to-high level of support	84 (66.7)	42 (33.3)	16.74	<0.001 *	0.32
Limited or no support	12 (30.0)	28 (70.0)			
**Received sufficient support from Government of Canada since the COVID-19 pandemic declared**					
Some-to-high level of support	34 (73.9)	12 (26.1)	7.96	<0.01 *	0.22
Limited or no support	58 (49.6)	59 (50.4)			
**Received sufficient support from Government of Alberta since the COVID-19 pandemic declared**					
Some-to-high level of support	27 (71.1)	11 (28.9)	2.37	0.18	0.13
Limited or no support	62 (56.9)	47 (43.1)			
**Received sufficient support from the employer since the COVID-19 pandemic declared**					
Some-to-high level of support	77 (63.6)	44 (36.4)	7.49	0.01	0.21
Limited or no support	18 (40.0)	27 (60.0)			

** Fisher’s exact test. * *p* < 0.05.

**Table 3:** Logistic regression model for respondents’ likelihood to present with moderate-to-severe anxiety.

Characteristics	B	S.E.	Wald	df	*p* Value	Odd’s Ratio	95% C.I. for Odd’s Ratio	
							Lower	Upper
**Not employed**	2.046	1.327	2.375	1	0.123	7.734	0.574	104.303
**Having received depression diagnosis from a health professional**	0.799	0.545	2.149	1	0.143	2.223	0.764	6.468
**Having received anxiety diagnosis from a health professional**	0.870	0.561	2.411	1	0.121	2.388	0.796	7.166
**Have received mental health counselling in the past year**	0.594	0.534	1.241	1	0.265	1.812	0.637	5.158
**Would like to receive mental health counselling**	1.285	0.482	7.118	1	0.008 *	3.616	1.406	9.296
**Not on any medication for mental health concerns**	−0.485	0.616	0.619	1	0.431	0.616	0.184	2.061
**Job loss due to the COVID-19 pandemic (Yes)**	1.482	0.753	3.876	1	0.049 *	4.401	1.007	19.241
**Received limited or no family support since the COVID-19 pandemic declared**	1.458	0.546	7.124	1	0.008 *	4.296	1.473	12.530
**Received limited or no support from Government of Canada since the COVID-19 pandemic declared**	0.812	0.524	2.400	1	0.121	2.253	0.806	6.292
**Received limited or no support from the employer since the COVID-19 pandemic declared**	−0.077	0.477	0.026	1	0.871	0.925	0.363	2.358
**Constant**	−2.922	0.584	24.993	1	0.000	0.054		

* Significance at *p* < 0.05. C.I.: confidence interval. S.E.: standard error. Df: degree of freedom.

## Data Availability

Data is available upon reasonable request to the submitting author.
